# Unresolved Issues in Implementing Integrated Management of Childhood Illness (IMCI) Approach

**DOI:** 10.34172/ijhpm.2020.89

**Published:** 2020-06-07

**Authors:** Masahiro J. Morikawa

**Affiliations:** Department of Family Medicine, University of Virginia, Charlottesville, VA, USA.

## Dear Editor,

Debate has been continuing for the implementation for Integrated Management of Childhood Illnesses (IMCI) strategy on childhood pneumonia to curve under-5 mortality in low- and middle-income countries (LMICs). Recent systematic reviews are still divided into both positives^1,2^ and negatives^3^ on the effectiveness of training and supportive supervision.^4^ IMCI approach itself has been going through several revisions in clinical signs^5^ and more recent evidences came out to suggested none of the clinical signs were sufficiently reliable to diagnose childhood pneumonia.^6^ Current debates are how to employ point-of-care pulse oximetry (Pox) in IMCI approach to improve the detection of hypoxia.^7^ The new technology itself, however, is not necessarily a panacea: Literature^8^ showed low current knowledge and usage of Pox at both district hospitals and healthcare posts.^9^ And my recent observation^10^ concurs the argument that Pox itself needs to be revised as a reliable, easy to use device at the bedside.^11^ All of these discussions, mixed results on training in LMICs, the development of new technology, and my recent observations, are all reminiscent of the fact that IMCI approach for childhood pneumonia is at chasm.

More than 10 years ago, we looked at the referral pattern and the initial care seeking behavior among remote six districts in Quetzaltenango, Guatemala where we implemented IMCI training among community healthcare workers recruited by the government for two consecutive years.

We interviewed family members of deceased children in the project area to figure out where they first looked for help. Less than fifth of them (17% in 2006 and 12% in 2007) sought initial care in government-run healthcare facilities. In fact, in both years, more people visited private clinics or pharmacies when their children were seriously ill. It was not surprising to find a significant under-utilization of the public healthcare resources in LMICs^12^ but we wondered why so low despite the free care. Competitive and parallel healthcare providers (non-governmental agencies, pharmacies, traditional healers and private clinics) eventually undermines the appropriate allocation of limited healthcare resources in LMICs and that will further damage the trust among the people towards the public health system.^13^

We then looked at the number of pneumonia referral cases from the first level of care to referral facilities by the IMCI algorithm. The proportion of patients who followed the referral and actually sought care at referral facilities was far less than the referred cases (37% in 2006 and 17% in 2007), the overall under-5 mortality in the area, however, did not show any differences in these two years.

Referral from the first level of care to the higher tier facility has been recognized as a problem since the inception of IMCI approach^14^ due to the low specificity of the clinical signs in the algorithm to diagnose presumptive pneumonia and often ended up over-referral.^15^ Our finding supported the low specificity of the algorithm-based care with IMCI approach. Over-referral in a community where transportation is a challenge, such as in remote mountainous village in Guatemala, would eventually become an issue of a trust to the healthcare system if it happens repeatedly.

I have no doubt that the development of reliable, easy to use point-of-care Pox and effective training to operate the device will play a key role in improving the care for childhood pneumonia: What we should not forget is though we have to make sure that the future revisions and new innovations for IMCI should help gaining the trust from the community towards public healthcare system as well as healthcare providers’ confidence in making diagnoses and referrals.

## Ethical issues

Not applicable.

## Competing interests

Author declares that he has no competing interests.

## Author’s contribution

MJM is the single author of the paper.
